# Tumor-Associated CD68^+^, CD163^+^, and MARCO^+^ Macrophages as Prognostic Biomarkers in Patients With Treatment-Naïve Gastroesophageal Adenocarcinoma

**DOI:** 10.3389/fonc.2020.534761

**Published:** 2020-10-23

**Authors:** Martin Jeremiasen, David Borg, Charlotta Hedner, Maria Svensson, Björn Nodin, Karin Leandersson, Jan Johansson, Karin Jirström

**Affiliations:** ^1^Department of Clinical Sciences Lund, Surgery, Lund University, Lund, Sweden; ^2^Skåne University Hospital, Lund, Sweden; ^3^Department of Clinical Sciences Lund, Oncology and Therapeutic Pathology, Lund University, Lund, Sweden; ^4^Cancer Immunology, Department of Translational Medicine, Lund University, Malmö, Sweden

**Keywords:** esophageal cancer, gastric cancer, macrophages, prognosis, treatment naïve

## Abstract

**Background:** Despite improvements in surgical methodologies and perioperative chemo- and radiotherapy, the prognosis for patients with esophageal and gastric cancer remains poor. Hence, there is a great need to identify complementary biomarkers for improved treatment stratification. Tumor-infiltrating immune cells have been shown to impact on outcome in many types of cancer, including gastroesophageal cancer. The aim of this present study was to examine the prognostic value of tumor-infiltrating macrophages in gastroesophageal adenocarcinoma.

**Methods:** The density of CD68^+^, CD163^+^, and MARCO^+^ macrophages was assessed by immunohistochemistry on tissue microarrays with primary tumors from a consecutive, retrospective cohort of 174 patients with treatment-naïve gastroesophageal adenocarcinoma. Total densities and infiltration in tumor nest (TN) were denoted as none/sparse (0), intermediate (1), or high (2). The impact on overall survival (OS) was examined by Kaplan–Meier analysis, log-rank test, and Cox proportional hazards modeling.

**Results:** Increased infiltration of both CD68^+^ and CD163^+^, but not MARCO^+^, macrophages in TN was significantly associated with a stepwise reduced survival. Median OS for patients with none/sparse, intermediate, and high CD68^+^ TN infiltration was 4.4, 2.6, and 1.0 years, respectively. Median OS for patients with none/sparse, intermediate, and high CD163^+^ TN infiltration was 4.4, 2.2, and 1.1 years, respectively. High infiltration of CD68^+^ macrophages remained an independent prognostic factor in adjusted analysis (hazard ratio = 1.61, 95% confidence interval = 1.02–2.55, and *p* = 0.041).

**Conclusion:** Infiltration of CD68^+^ and CD163^+^, but not MARCO^+^, macrophages is prognostic for OS in gastroesophageal adenocarcinoma. The relevance of this finding in clinical practice remains to be elucidated.

## Introduction

Cancer of the esophagus and stomach are both leading causes of cancer-related mortality around the world. Most patients with tumors in these locations are not eligible for curative treatment due to disseminated disease at the time of diagnosis or poor performance status. Hence, the prognosis for 5-year survival is no more than 15–20% in Western populations (1).

Prognosis after treatment with curative intention, typically involving a combination of surgery and oncological therapy, varies depending on several well-known risk factors such as residual tumor (R–) status and pathological stage (2, 3). To better understand the heterogeneity of gastroesophageal (GE) cancer and to predict clinical outcome, research has also focused on a multitude of investigative biomarkers, but to date none has been incorporated into clinical practice. The importance of the tumor microenvironment (TME) and the immune system for cancer progression has gained increasing attention in recent years. High infiltration of immune cells of the T and B lineage in the TME has been shown to be associated with a prolonged survival in esophageal and gastric adenocarcinoma (4–6). Macrophages are part of the innate immune system but are also involved in the process of initiating specific immune responses to pathogens, i.e., adaptive immunity. By virtue of the plasticity of macrophages harboring both anti- (M1-type) and pro-tumoral (M2) properties (7), findings regarding their prognostic significance in a variety of cancer forms are somewhat conflicting.

High infiltration of tumor-associated macrophages (TAMs) in tumor stroma but not in tumor nest was found to be associated with more aggressive tumors and decreased overall survival in breast cancer patients (8). Cui et al. (9) found an association between the M2/M1-ratio and liver metastasis in colorectal cancer. Conversely, another study indicated a prolonged survival for patients with colorectal liver metastases with high infiltration of TAMs (10).

In patients with adenocarcinoma of the esophagus, a high M2/M1-ratio was associated with poor survival and lymph node metastasis (11), and in patients with squamous cell carcinoma (SCC) of the esophagus, a high infiltration of M2 macrophages was associated with worse survival and poor response to chemotherapy (12). In gastric cancer, high infiltration of M2 macrophages was associated with poor prognosis (13), and high infiltration of CD68^+^ TAMs was associated with clinical stage and poor outcome (14).

The aim of this study was to investigate the prognostic impact of different subsets of TAMs in a previously well-described cohort of 174 consecutive patients with resected treatment-naïve GE adenocarcinoma.

## Materials and Methods

### Study Design and Participants

The study cohort comprised 174 consecutive patients treated surgically for esophageal or gastric adenocarcinoma between 1 Jan 2006 and 31 Dec 2010 in the University hospitals of Lund and Malmö, Sweden. The standard surgical procedure for esophageal cancers (C15–C16.0B) was the two field Ivor Lewis esophagectomy with reconstruction by means of a gastric tube. The standard surgical procedure for distal gastric cancer was the Billroth II-procedure whereas patients with more proximal or diffuse tumor types were resected with a total abdominal gastrectomy and reconstruction with a Roux-loop. In some patients with large tumors at the gastroesophageal junction, an extended gastrectomy was performed. In patients with locally advanced disease, additional resection of the spleen and/or colon was performed when indicated. The lymph node clearance always included a D1+ or D2-dissection. Tumor stage was classified according to TNM 8, Siewert I and II tumors being classified as esophageal tumors (C15.0A–C16.0B) and Siewert III tumors as gastric tumors (C16.0C–C16.9) (15). Ninety-nine patients were classified as having esophageal cancer and 75 patients as having gastric cancer. No patients received any neoadjuvant treatment before surgery. Only 13 patients received adjuvant treatment. Eleven patients received fluoropyrimidine-based chemoradiotherapy, typically to 40 or 45 Gray (Gy). One patient received radiotherapy alone and another patient received chemotherapy alone. The cohort has been described in several previous studies (4, 16, 17). Clinical data regarding recurrence and vital status were obtained retrospectively from medical records, and the last update, with additional re-examination of some of the clinicopathological data, was performed in March 2016. Residual tumor status (R-status) was defined as R0, no residual tumor; R1, possible microscopic residual tumor (within 1 mm of the resection margin); and R2, macroscopic residual tumor.

The study was approved by the Regional Ethics committee of Lund (ref no 445/07), whereby no need for consent other than the option to opt-out was waived.

### Tissue Microarray Construction, Immunohistochemistry (IHC), and Staining Evaluation

Tissue microarrays (TMAs) were constructed using a semi-automated arraying device (TMArrayer, Pathology Devices, Westminister, MD, USA). Duplicate tissue cores were obtained from primary tumors, each from a separate donor block. For immunohistochemical (IHC) analysis of expression of the pan-macrophage marker CD68, the M2 macrophage marker CD163, and the scavenger receptor macrophage receptor with collagenous structure (MARCO), 4 μm TMA-sections were pre-treated using the DAKO PT link system (DAKO; Glostrup, Copenhagen, Denmark) and stained in an Autostainer Plus (DAKO; Glostrup, Copenhagen, Denmark) with the following antibodies: CD68: clone KP1, diluted 1:1000, Dako, Glostrup, Denmark, CD163: clone 10D6 diluted 1:200, Novus Biologicals, Abingdon, United Kingdom, MARCO clone HPA063793, diluted 1:250, Atlas Antibodies, Bromma, Sweden. All stainings were evaluated by two independent observers (MJ and KJ), one being a senior pathologist (KJ). Both observers were blinded to clinical and outcome data. The total infiltration as well as infiltration into tumor nest (TN), defined as being juxtaposed to a tumor cell or in the direct vicinity of a tumor cell, was denoted as 0 (none), 1 (sparse), 2 (intermediate), or 3 (high) in accordance with a previous study (8). However, as only a few cases fell into the negative category, three categories were used for statistical purposes: 0 (negative/sparse), 1 (intermediate), and 2 (high). In cases with differing expression between the two cores the one with the highest score was used.

### Statistical Analysis

For analysis of differences in the distribution of CD68, CD163, and MARCO expression according to clinicopathological parameters, the non-parametric Mann–Whitney *U*-test was used for continuous variables and the Chi-squared test was used for categorical variables. Kaplan–Meier analysis and the log rank test were used to compare overall survival (OS) in patients with IHC staining 0–1 vs. 2. Unadjusted and adjusted hazard ratios (HR) for OS were calculated using Cox regression proportional hazard modeling. The adjusted model only included those variables who were significant in the unadjusted model. The Backward conditional model according to Wald was used in the adjusted model. All tests were two sided and *p* < 0.05 were considered significant. All statistical analyses were performed using IBM SPSS Statistics version 25 (SPSS Inc., Chicago, IL, USA).

## Results

### Infiltration of CD68^+^, CD163^+^, and MARCO^+^ Macrophages in Primary Tumors

**Figure 1:**
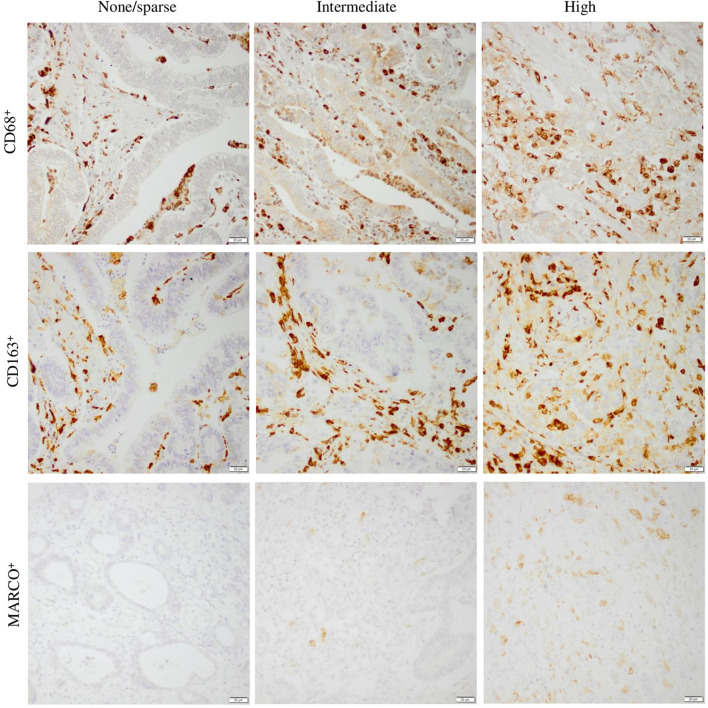
Sample immunohistochemical images (20× magnification) representing different categories of CD68, CD163, and MARCO infiltration into tumor nest, ranging from none/sparse to high. Scale bar = 20 μm.

Due to loss of material or low tumor content, the expression of CD68 could be evaluated in 162/174 (93.1%) of the total group of patients. The expression of CD163 could be evaluated in 165/174 (94.8%) of the total group of patients, whereas the expression of MARCO could be evaluated in 166/174 (95.4%) cases. Sample IHC images of staining for CD68, CD163, and MARCO are shown in Figure 1. Notably, the infiltration of MARCO^+^ macrophages was considerably more sparse than the infiltration CD68^+^ and CD163^+^ macrophages.

### Prognostic Significance of CD68^+^, CD163^+^, and MARCO^+^ Macrophage Infiltration in the Primary Tumors

**Figure 2:**
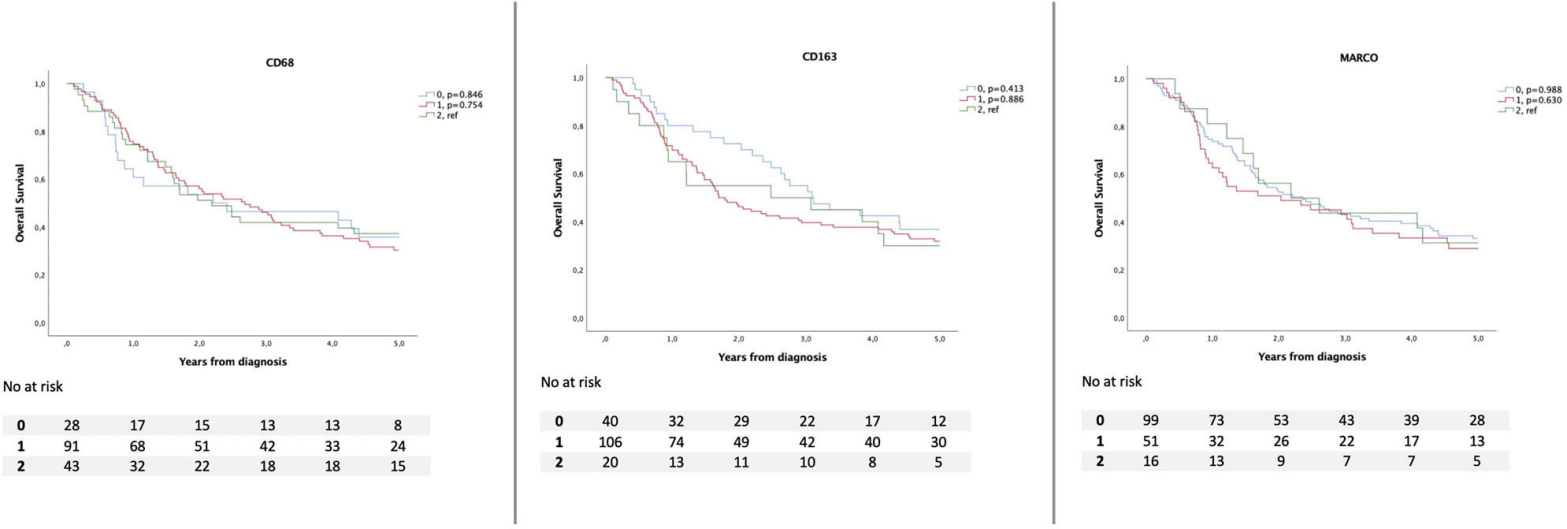
Kaplan–Meier estimates of overall survival according to total infiltration of CD68^+^, CD163^+^, and MARCO^+^ macrophages.

**Figure 3:**
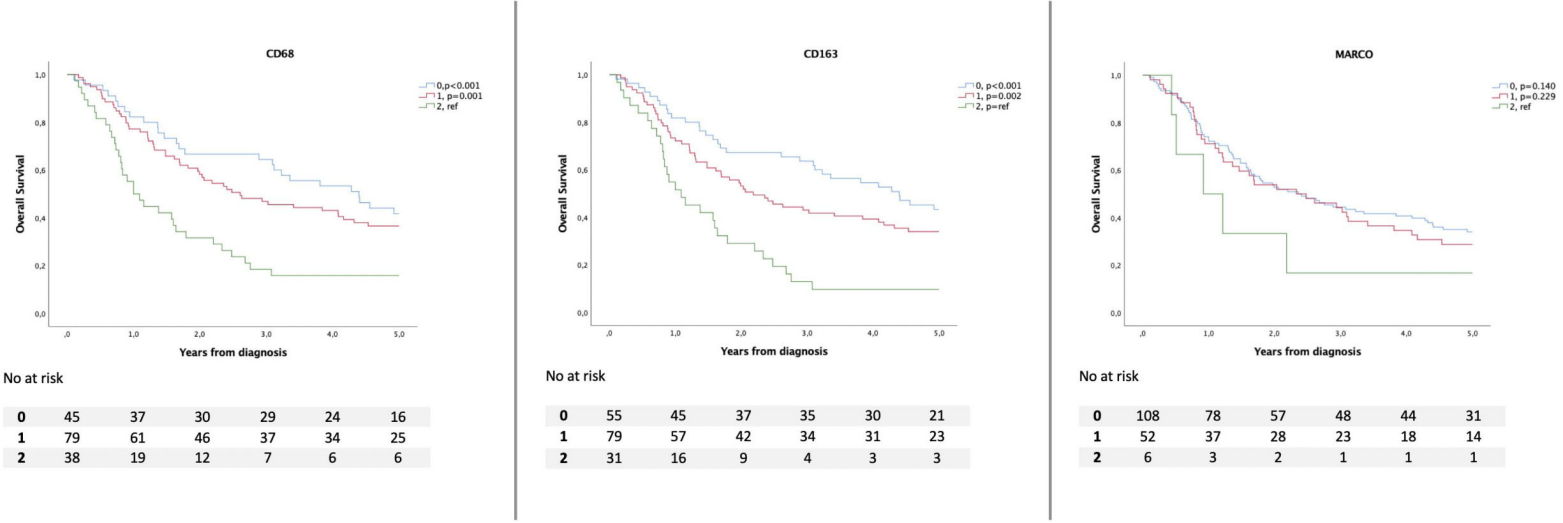
Kaplan–Meier estimates of overall survival according to infiltration into tumor nest of CD68^+^, CD163^+^, and MARCO^+^ macrophages.

Kaplan–Meier analyses of OS in relation to total and TN infiltration of CD68^+^, CD163^+^, and MARCO^+^ macrophages, respectively, are shown in Figures 2, 3, whereby infiltration in TN was demonstrated to confer a stronger prognostic value, in a stepwise fashion (Figure 3). There was no significant association between the expression of MARCO and OS.

Based on visual inspection of the Kaplan–Meier curves, CD68^+^, CD163^+^, and MARCO^+^ infiltration was dichotomized into none/sparse and intermediate (0–1) vs. high expression (2). Associations between the high infiltration of CD68^+^ and CD163^+^ in TN and a reduced OS were confirmed in unadjusted Cox regression analysis (HR = 2.27; 95% CI = 1.49–3.44, *p* < 0.001 and HR = 2.49; 95% CI = 1.61–3.85, *p* < 0.001; Table 1). In the adjusted model, only incorporating variables that were significant in the unadjusted model (age, pStage, differentiation grade, R-status, CD68, and CD 163), the following factors remained significant: High CD68^+^ infiltration in TN (HR = 1.61; 95% CI = 1.02–2.55, *p* = 0.041), age, pStage, and R-status. The associations between CD68^+^ and CD163^+^ macrophage infiltration with OS were also confirmed in an unadjusted and adjusted model exchanging pathological stage for clinical stage (data not shown). Subgroup Cox regression analysis in patients with esophageal cancer showed that high infiltration of CD68^+^ macrophages was prognostic in the unadjusted model whereas high infiltration of both CD68^+^ and CD163^+^ macrophages were prognostic in gastric cancer (Supplementary Tables 1, 2). However, none of these associations was confirmed in the adjusted model. MARCO was not prognostic in any of the subgroup analyses.

**Table 1:** Hazard ratio for death according to clinicopathological factors, CD68^+^, CD163^+^, and MARCO^+^ macrophages in 174 patients with esophagogastric cancer.

	**Overall survival total cohort**				
	***n***	**Unadjusted HR (95% CI)**	***p*-value**	**Adjusted HR (95% CI)**	***p*-value**
**Age**			0.001		<0.001
Continuous	174	1.03 (1.01–1.05)		1.04 (1.02–1.05)	
**Gender**			0.149		
Female	39	1.00		–	–
Male	135	0.73 (0.48–1.12)		–	–
**Tumor stage**			<0.001		<0.001
1	26	1.00		1.00	
2	41	1.72 (0.76–3.91)		2.02 (0.69–5.96)	
3	56	3.78 (1.77–8.05)		4.88 (1.70–13.95)	
4	51	5.94 (2.78–12.70)		7.52 (2.60–21.79)	
**Differentiation Grade**			0.010		0.06
High/moderate	61	1.00		1.00	
Low	113	1.70 (1.13–2.55)		1.53 (0.98–2.37)	
**Location**			0.463		
Esophagus + SI–II	99	1.00		–	–
SIII+Stomach	75	1.15 (0.80–1.66)		–	–
**R-status**			<0.001		0.003
R0	122	1.00		1.00	
R1–R2	52	2.81 (1.92–4.12)		1.89 (1.25–2.88)	
**CD68**			<0.001		0.041
Low (0–1)	124	1.00		1.00	
High (2)	38	2.27 (1.49–3.44)		1.61 (1.02–2.55)	
**CD163**			<0.001		0.46
Low (0–1)	134	1.00		1.00	
High (2)	31	2.49 (1.61–3.85)		0.68 (0.25–1.87)	
**MARCO**			0.162		
Low (0–1)	160	1.00		–	–
High (2)	6	1.90 (0.77–4.67)		–	–
**Adjuvant treatment**			0.894		
Yes	13	1.05 (0.53–2.07)		–	–
No	161	1.00		–	–

*R0, no residual tumor; R1, possible microscopic residual tumor (within 1 mm of the resection margin); R2, macroscopic residual tumor. SI–III, Siewert I–III*.

### Associations of CD68^+^, CD163^+^, and MARCO^+^ Macrophage Infiltration With Clinicopathological Parameters

The associations of CD68^+^, CD163^+^, and MARCO^+^ macrophage infiltration into TN with patient and tumor characteristics are shown in Table 2. High infiltration of CD68^+^ and CD163^+^ macrophages into TN was associated with higher pT-stage (*p* = 0.02 and 0.009, respectively), low differentiation grade (*p* < 0.001 for both), diffuse tumor type (*p* < 0.001 for both), and infiltration of both CD68^+^ and CD163^+^ macrophages were higher in the stomach compared to the esophagus (*p* = 0.015 and 0.003, respectively). High infiltration of CD68^+^, but not CD163^+^, macrophages was associated with higher pN-stage (*p* = 0.031). Of note, there were no significant associations of any macrophage subsets with mismatch repair deficiency (dMMR), but CD163^+^ macrophage infiltration was positively associated with expression of programmed death ligand 1 (PD-L1) on tumor cells (*p* = 0.035), and MARCO^+^ density was positively associated with PD-L1 expression on both tumor cells (*p* = 0.009) and immune cells (*p* = 0.025). The infiltration of CD68^+^ and CD163^+^ macrophages was strongly intercorrellated (*p* < 0.001), but there were no significant associations between MARCO^+^ macrophages and the other investigated macrophage subsets.

**Table 2:** Associations of CD68, CD163, and MARCO expression with clinicopathological characteristics.

**Factor**	**CD68 TN infiltration**				**CD163 TN infiltration**				**MARCO TN infiltration**			
***n* (%)**	**0**	**1**	**2**	***P***	**0**	**1**	**2**	***P***	**0**	**1**	**2**	***P***
	45 (25.9)	79 (45.4)	38 (21.8)		55 (31.6)	79 (45.4)	31 (17.8)		108 (62.1)	52 (29.9)	6 (3.4)	
Age				0.314				0.366				0.750
mean, median (range)	70.1, 69.2 (48.2–88.8)	69.4, 70.9 (42.6–88.5)	72.6, 74.3 (48.7–94.4)		69.0, 68.7 (48.2–88.8)	70.2, 72.2 (42.6–88.5)	72.5, 72.6 (53.2–94.4)		69.8, 70.6 (48.2–88.6)	71.1, 69.0 (42.6–94.4)	69.2, 73.6 (50.2, 78.6)	
Gender				0.500				0.249				0.908
Female	9 (20.0)	17 (21.5)	10 (26.3)		9 (16.4)	20 (25.3)	8 (25.8)		25 (23.1)	11 (21.2)	2 (33.3)	
Male	36 (80.0)	62 (78.5)	28 (73.7)		46 (83.6)	59 (74.7)	23 (74.2)		83 (76.9)	41 (78.8)	4 (66.7)	
pT-stage				0.020				0.009				0.576
T1	6 (13.3)	6 (7.6)	1 (2.6)		6 (10.9)	7 (8.9)	0 (0.0)		10 (9.3)	3 (5.8)	0 (0.0)	
T2	9 (20.0)	15 (19.0)	6 (15.8)		11 (20.0)	15 (19.0)	4 (12.9)		18 (16.7)	11 (21.2)	1 (16.7)	
T3	26 (57.8)	47 (59.5)	22 (57.9)		32 (58.2)	46 (58.2)	17 (54.8)		62 (57.4)	31 (59.6)	3 (50.0)	
T4	4 (8.9)	11 (13.9)	9 (23.7)		6 (10.9)	11 (13.9)	10 (32.3)		18 (16.7)	7 (13.5)	2 (33.3)	
pN-stage				0.031				0.112				0.391
N0	15 (33.3)	27 (34.2)	8 (21.1)		17 (30.9)	26 (32.9)	8 (25.8)		37 (34.3)	14 (26.9)	1 (16.7)	
N1	11 (24.4)	13 (16.5)	5 (13.2)		14 (25.5)	12 (15.2)	4 (12.9)		16 (14.8)	13 (25.0)	0 (0.0)	
N2	13 (28.9)	15 (19.0)	12 (31.6)		16 (29.1)	16 (20.3)	9 (29.0)		26 (24.1)	13 (25.0)	2 (33.3)	
N3	6 (13.3)	24 (30.4)	13 (34.2)		8 (14.5)	25 (31.6)	10 (32.3)		29 (26.9)	12 (23.1)	3 (50.0)	
pM-stage				0.938				0.544				0.562
M0	39 (86.7)	72 (91.1)	33 (86.8)		48 (87.3)	73 (92.4)	25 (80.6)		94 (87.0)	48 (92.3)	5 (83.3)	
M1	6 (13.3)	7 (8.9)	5 (13.2)		7 (12.7)	6 (7.6)	6 (19.4)		14 (13.0)	4 (7.7)	1 (16.7)	
Differentiation grade				0.001				0.001				0.478
Low	21 (46.7)	57 (72.2)	30 (78.9)		26 (47.3)	57 (72.2)	26 (83.9)		74 (68.5)	32 (61.5)	4 (66.7)	
High/moderate	24 (53.3)	22 (27.8)	8 (21.1)		29 (52.7)	22 (27.8)	5 (16.1)		34 (31.5)	20 (38.5)	2 (33.3)	
Residual tumor status				0.290				0.181				0.692
R0	35 (77.8)	54 (68.4)	22 (57.9)		41 (74.5)	53 (67.1)	17 (54.8)		71 (65.7)	36 (69.2)	4 (66.7)	
R1	5 (11.1)	24 (30.4)	14 (36.8)		9 (16.4)	25 (31.6)	11 (35.5)		31 (28.7)	13 (25.0)	2 (33.3)	
R2	5 (11.1)	1 (1.3)	2 (5.3)		5 (9.1)	1 (1.3)	3 (9.7)		6 (5.6)	3 (5.8)	0 (0.0)	
Location				0.015				0.003				0.149
Esophagus	31 (68.9)	46 (58.2)	16 (42.1)		39 (70.9)	43 (54.4)	12 (38.7)		56 (51.9)	33 (63.5)	4 (66.7)	
Stomach	14 (31.1)	33 (41.8)	22 (57.9)		16 (29.1)	36 (45.6)	19 (61.3)		52 (48.1)	19 (36.5)	2 (33.3)	
Laurén				<0.001				<0.001				0.050
Intestinal	42 (93.3)	53 (67.1)	16 (42.1)		52 (94.5)	50 (63.3)	11 (35.5)		67 (62.0)	42 (80.8)	4 (66.7)	
Mixed	0 (0.0)	3 (3.8)	6 (15.8)		0 (0.0)	4 (5.1)	5 (16.1)		6 (5.6)	3 (5.8)	0 (0.00)	
Diffuse	3 (6.7)	23 (29.1)	16 (42.1)		3 (5.5)	25 (31.6)	15 (48.4)		35 (32.4)	7 (13.5)	2 (33.3)	
MMR status				0.860				0.965				0.192
pMMR	42 (93.3)	71 (89.9)	36 (94.7)		51 (92.7)	72 (91.1)	29 (93.5)		101 (93.5)	46 (88.5)	5 (83.3)	
dMMR	3 (6.7)	8 (10.1)	2 (5.3)		4 (7.3)	7 (8.9)	2 (6.5)		7 (6.5)	6 (11.5)	1 (16.7)	
PD-L1 expression in TC				0.070				0.035				0.009
<1%	40 (90.9)	55 (70.5)	26 (72.2)		49 (90.7)	54 (69.2)	21 (72.4)		86 (81.1)	36 (70.6)	3 (50.0)	
1–49%	4 (9,1)	18 (23.1)	10 (27.8)		5 (9.3)	19 (24.4)	8 (27.6)		19 (17.9)	12 (23.5)	1 (35.5)	
≥50%	0 (0)	5 (6.4)	0 (0)		0 (0)	5 (6.4)	0 (0)		1 (0.9)	3 (5.9)	1 (16.7)	
PD-L1 expression in IC				0.481				0.449				0.025
<10%	32 (72.7)	36 (46.2)	23 (63.9)		38 (70.4)	37 (47.4)	19 (65.5)		70 (66.0)	22 (43.1)	3 (50.0)	
10–49%	9 (20.5)	35 (44.9)	11 (30.6)		13 (24.1)	33 (42.3)	9 (31.9)		30 (28.3)	23 (45.1)	3 (50.0)	
≥50%	3 (6.8)	7 (9.0)	2 (5.6)		3 (5.6)	8 (10.3)	1 (3.4)		6 (5.7)	6 (11.8)	0 (0)	
CD68 TN cat								<0.001				0.648
Low	–	–	–		45 (83.3)	0 (0.0)	0 (0.0)		31 (29.5)	13 (26.0)	0 (0.0)	
Intermediate	–	–	–		9 (16.7)	70 (89.7)	0 (0.0)		48 (45.7)	26 (52.0)	5 (83.3)	
High	–	–	–		0 (0.0)	8 (10.3)	30 (100.0)		26 (24.8)	11 (22.0)	1 (16.7)	
CD163 TN cat				<0.001								0.338
Low	45 (100)	9 (11.4)	0 (0)		–	–	–		38 (35.5)	16 (31.4)	0 (0.0)	
Intermediate	0 (0.0)	70 (88.6)	8 (21.1)		–	–	–		49 (45.8)	25 (49.0)	5 (83.3)	
High	0 (0.0)	0 (0.0)	30 (78.9)		–	–	–		20 (18.7)	10 (19.6)	1 (16.7)	
MARCO TN cat				0.648				0.338				
Low	31 (70.5)	48 (60.8)	26 (68.4)		38 (70.4)	49 (62.0)	20 (64.5)		–	–	–	
Intermediate	13 (29.5)	26 (32.9)	11 (28.9)		16 (29.6)	25 (31.6)	10 (32.3)		–	–	–	
High	0 (0.0)	5 (6.3)	1 (2.6)		0 (0.0)	5 (6.3)	1 (3.2)		–	–	–	

*N1, metastasis in 1–2 regional lymph nodes; N2, metastasis in 3–6 regional lymph nodes; N3, metastasis in seven or more regional lymph nodes; R0, no residual tumor; R1, possible microscopic residual tumor (within 1 mm of the resection margin); R2, macroscopic residual tumor; PD-L1, programmed death ligand 1; TC, tumor cells; IC, immune cells*.

The associations between TAMs and other previously examined tumor-infiltrating immune cells (4, 5) were also investigated. This revealed significant inverse associations between CD68^+^ and CD163^+^ TAMs and CD8^+^ T cells, as well as between MARCO^+^ TAMs and CD20^+^ B cells. There were no significant associations between any of the investigated macrophage subsets and FoxP3^+^ T cells or NKp46^+^ NK-cells (data not shown).

## Discussion

The results of this study demonstrate that high infiltration of CD68^+^ and CD163^+^ macrophages in TN were negative prognostic factors for OS in this cohort of 174 consecutive patients with treatment-naïve GE adenocarcinoma. Both high infiltration of CD68^+^ and CD163^+^ macrophages were significantly associated with several established unfavorable clinicopathological factors. Yet, high infiltration of CD68^+^ macrophages remained an independent prognostic factor for shorter OS in the adjusted analysis, together with other well-known significant factors such as pStage, R-status, and age.

The majority of studies evaluating the prognostic value of TAMs in esophageal cancer is based on patients with SCC, not adenocarcinoma as in the present study. In a Japanese study, high infiltration of TAMs, especially CD163^+^ macrophages, in patients with esophageal SCC was associated with worse prognosis and poor response to neoadjuvant treatment (12), whereas Yagi et al. (18) argued for the negative prognostic role of high infiltration of CD163^+^ and CD204^+^ macrophages in esophageal cancer in a cohort of 305 patients who had undergone esophageal resection, of whom over 90% had SCC. Both of these studies included patients with neoadjuvant treatment to various extent in their protocols. Another study showed a relationship between CD204^+^ macrophages and more aggressive tumors in patients not subjected to neoadjuvant oncological treatment with SCC (19).

One of the few studies addressing the role of TAMs in esophageal adenocarcinoma is the study of Cao et al. (11) that showed a correlation between the ratio of M2/M1-macrophages with lymph node metastasis and poor survival in a series of patients who had undergone esophageal resection, where 20/53 (38%) of patients had received neoadjuvant treatment. Our results are in line with these findings, even though our subgroup analyses failed to show significant association with survival for CD68^+^ and CD163^+^ TAMs in patients with esophageal and gastric cancer separately, most likely due to the analyses being underpowered.

Further support of our results regarding the role of TAMs in gastric cancer was given by Zhang et al. (20) who showed that high infiltration of CD68^+^ macrophages was associated with aggressive features and worse survival in 178 gastric cancer patients. Also, in an analysis of the expression of CD68^+^ macrophages in a series of 401 patients operated on for gastric cancer, another group found an association between CD68-positive tumors and shorter disease-free survival (21). Both studies included only patients without prior oncological treatment before surgery.

Infiltration of the more conspicuous subset of MARCO^+^ macrophages was not a prognostic factor for OS in our study. The literature to date on the prognostic significance of MARCO in cancer is sparse. Sun et al. (22) reported that a decreased expression of MARCO was associated with tumor progression and poor prognosis in hepatocellular carcinoma and Lundgren et al. (23) showed that high density of MARCO^+^ TAMs in certain subtypes of periampullary pancreatic cancer predicted shorter survival. To the best of our knowledge, the expression and prognostic impact of MARCO in GE adenocarcinoma has not yet been reported.

Our results, indicating a prognostic significance of high infiltration of CD68^+^ and CD163^+^ macrophages in tumor nest, rather than the total infiltration, highlight the importance of considering the compartmental localization of TAMs in the TME. Medrek et al. (8) showed that high infiltration of CD68^+^ macrophages in tumor stroma but not in TN was associated with reduced breast cancer specific survival, and Ohno et al. (24) also stressed the importance of the histological location of infiltrating TAMs in endometrial cancer as a prognostic tool.

Translating the results from our cohort of treatment-naïve GE adenocarcinoma into clinical practice, high infiltration of in particular CD68^+^ but also CD163^+^ macrophages could possibly warrant intensified adjuvant treatment for patients who are at higher risk for recurrent disease after surgery without prior oncological treatment. Notably, to the best of our knowledge, this is the largest study to date to include a well-defined consecutive series of treatment-naïve esophageal adenocarcinoma patients, thus being as ideal as can be for prognostic biomarker studies in the retrospective setting. Therefore, the results from this study support the hypothesis that high infiltration of CD68^+^ and CD163^+^ macrophages in pre-operative biopsies signify more aggressive disease. Importantly, multivariable analysis, also when exchanging pathological stage for clinical stage, showed that high infiltration of CD68^+^ macrophages was still an independent prognostic factor for worse OS. Future prospective studies with analysis of TAM-infiltration in pre-operative biopsies will have to address the relevance of this hypothesis in the clinical setting. In analogy with the study of Sugimura et al. (12) on SCCs, high infiltration of M2 macrophages in biopsies from patients undergoing neoadjuvant treatment for esophageal and gastric adenocarcinomas could hypothetically signal weak therapeutic response to the oncological treatment, thus indicating a possible need for change of treatment strategy.

The dichotomized cutoff used in this study is in line with a previous paper on breast cancer (8), but it should be pointed out that there was indeed a stepwise increased negative prognostic impact for the three original scoring categories. Additional studies are required to determine which cut-off points will provide the most accurate prognostic or predictive information for different macrophage subsets. To this end, automated image analysis may also be a valuable tool.

Even though multimodality treatment has been shown to increase survival and is currently gold standard in the curative setting in most centers, a considerable proportion of patients with esophageal and gastric cancer is still being operated on without prior neoadjuvant oncological treatment (1). On the other hand, there is mounting evidence of certain subtypes of gastric tumors, such as dMMR tumors, being less sensitive to neoadjuvant treatment regimens (25, 26). To date, in some countries, pembrolizumab is the only approved immunotherapeutic agent in patients with unresectable, recurrent, or metastatic dMMR upper GI tumors after failure of initial systemic treatments. Ongoing studies will show whether immunotherapy might have a role also in the neoadjuvant or adjuvant setting (27). In the present study, there were no significant associations between any of the investigated macrophage subsets and dMMR-status. However, expression of programmed death ligand-1 (PD-L1) on both tumor cells and immune cells has previously been shown to be strongly associated with dMMR-status in the present cohort (17), and in this study, significant associations were observed between CD163^+^ macrophage infiltration and PD-L1 expression on tumor cells, and between MARCO^+^ macrophages and PD-L1 expression on both tumor cells and immune cells. Speculatively, these findings suggest that certain subsets of macrophages may be more relevant in the context of immunotherapy than for purposes of prognostication.

Finding biomarkers to predict response to oncological therapy is essential in the strive for more tailored and individual treatment regimens and has the potential to spare cancer patients the harm of unnecessary side effects as well as being cost-effective for the health care sector and society as a whole. The role of TAMs in this context is still investigational. In cancers where infiltrating innate immune cells clearly affect survival negatively, possible future combination strategies using antibodies targeting the innate immunosuppressive macrophages, such as M2-inhibitors, combined with check-point inhibition therapies affecting the adaptive immune response, might be a future way forward.

Of note, even though the TMA technique is a well-validated tool for biomarker studies (28), it comes with some limitations. The inherent risk of sampling bias was, however, reduced by taking duplicate cores from two different blocks of the primary tumor, and it must also be pointed out that even the use of full-face sections comes with a risk of sampling bias, since these also only represent a small fraction of the tumor. Another limitation is that the present study was not powered primarily for subgroup analysis and the results from these analyses must therefore be taken with some caution and validated in future studies. Neoadjuvant treatment, today gold standard in multimodality treatment of GE adenocarcinoma, was gradually introduced on a small scale in our department during the period of time when this study was conducted. These few patients were not included in this study, which might also be a potential weakness. On the other hand, all treatment-naïve patients included in this study were included consecutively, which minimizes the risk of selection bias.

In essence, this study shows that high infiltration of CD68^+^ macrophages into TN is an independent prognostic biomarker in patients with treatment-naïve resected GE adenocarcinomas. Future prospective studies will hopefully elucidate the potential role of TAMs not only in selecting patients who will benefit from neoadjuvant treatment, but also for prognostication after surgery in patients who have received neoadjuvant treatment.

## Data Availability Statement

The datasets generated for this study are available on request to the corresponding author.

## Ethics Statement

The studies involving human participants were reviewed and approved by Regional Ethics committee of Lund, Lund University, Sweden. Written informed consent for participation was not required for this study in accordance with the national legislation and the institutional requirements.

## Author Contributions

Conceived and designed the experiments: KJ. Performed the experiments: MJ, MS, BN, and KJ. Analyzed the data: MJ and KJ. Contributed reagents, materials, analysis tools: BN, CH, DB, KL, and JJ. Wrote the paper: MJ and KJ.

## Conflict of Interest

The authors declare that the research was conducted in the absence of any commercial or financial relationships that could be construed as a potential conflict of interest.
