# Supplementary material for: Tumor-Associated CD68+, CD163+, and MARCO+ Macrophages as Prognostic Biomarkers in Patients With Treatment-Naïve Gastroesophageal Adenocarcinoma
Source: Front Oncol. 2020 Oct 23;10:534761. doi: 10.3389/fonc.2020.534761 (PMC7645217; doi:10.3389/fonc.2020.534761)
Supplement: Supplementary file 1 [file Table_1.docx]

Supplement 1: Hazard ratio for death according to clinicopathological factors, CD68^+^, CD163^+^ and MARCO^+^ macrophages in esophageal cancer.

|  |  | Overall survival esophagus+SI-II | | | |
| --- | --- | --- | --- | --- | --- |
|  | **n** | **Unadjusted**  **HR (95% CI)** | **p-value** | **Adjusted**  **HR (95% CI)** | **p-value** |
| Age |  |  | 0.033 |  | 0.008 |
| Continuous | 99 | 1.03 (1.00-1.05) |  | 1.03 (1.01-1.06) |  |
| Gender |  |  | 0.034 |  | 0.168 |
| Female | 13 | 1.00 |  | 1.00 |  |
| Male | 86 | 0.49 (0.26-0.95) |  | 0.58 (0.27-1.26) |  |
| Tumor stage |  |  | <0.001 |  | 0.001 |
| 1 | 9 | 1.00 |  | 1.00 |  |
| 2 | 16 | 0.80 (0.19-3.36) |  | 0.28 (0.05-1.48) |  |
| 3 | 35 | 2.50 (0.75-8.29) |  | 1.26 (0.29-5.40) |  |
| 4 | 39 | 4.65 (1.42-15.21) |  | 2.05 (0.48-8.82) |  |
| Differentiation Grade |  |  | 0.022 |  | 0.09 |
| High/moderate | 42 | 1.00 |  | 1.00 |  |
| Low | 57 | 1.83 (1.09-3.06) |  | 1.59 (0.93-2.73) |  |
| R-status |  |  | <0.001 |  | 0.004 |
| R0 | 66 | 1.00 |  | 1.00 |  |
| R1-R2 | 33 | 2.64 (1.60-4.35) |  | 2.22 (1.30-3.79) |  |
| CD68 |  |  | 0.022 |  | 0.157 |
| Low (0-1) | 77 | 1.00 |  | 1.00 |  |
| High (2) | 16 | 2.05 (1.11-3.79) |  | 1.71 (0.82-3.57) |  |
| CD163 |  |  | 0.073 |  |  |
| Low (0-1) | 82 | 1.00 |  | ----- | ----- |
| High (2) | 12 | 1.86 (0.94-3.68) |  | ----- | ----- |
| MARCO |  |  | 0.547 |  |  |
| Low (0-1) | 89 | 1.00 |  | ----- | ----- |
| High (2) | 4 | 1.43 (0.45-4.56) |  | ----- | ----- |
| Adjuvant treatment |  |  | 0.745 |  |  |
| Yes | 8 | 0.86 (0.35-2.141) |  | ----- | ----- |
| No | 91 | 1.00 |  | ----- | ----- |
