# Supplementary material for: Tumor-Associated CD68+, CD163+, and MARCO+ Macrophages as Prognostic Biomarkers in Patients With Treatment-Naïve Gastroesophageal Adenocarcinoma
Source: Front Oncol. 2020 Oct 23;10:534761. doi: 10.3389/fonc.2020.534761 (PMC7645217; doi:10.3389/fonc.2020.534761)
Supplement: Supplementary file 2 [file Table_2.docx]

|  |  | Overall survival SIII-stomach | | | |
| --- | --- | --- | --- | --- | --- |
|  | **n** | **Unadjusted**  **HR (95% CI)** | **p-value** | **Adjusted**  **HR (95% CI)** | **p-value** |
| Age |  |  | 0.015 |  | 0.004 |
| Continuous | 75 | 1.03 (1.01-1.06) |  | 1.04 (1.01-1.07) |  |
| Gender |  |  | 0.895 |  |  |
| Female | 26 | 1.00 |  | ----- |  |
| Male | 49 | 1.04 (0.59-1.85) |  | ----- |  |
| Tumor stage |  |  | <0.001 |  | <0.001 |
| 1 | 17 | 1.00 |  | 1.00 |  |
| 2 | 25 | 2.64 (0.96-7.27) |  | 5.55 (1.26-24.46) |  |
| 3 | 21 | 7.02 (2.55-19.31) |  | 15.67 (3.45-71.13) |  |
| 4 | 12 | 11.35 (3.77-34.18) |  | 19.06 (3.63-100.20) |  |
| Differention Grade |  |  | 0.245 |  |  |
| High/moderate | 19 | 1.00 |  | ----- |  |
| Low | 56 | 1.49 (0.76-2.91) |  | ----- |  |
| R-status |  |  | <0.001 |  | 0.040 |
| R0 | 56 | 1.00 |  | 1.00 |  |
| R1-R2 | 22 | 3.47 (1.88-6.40) |  | 2..20 (1.04-4.68) |  |
| CD68 |  |  | 0.003 |  | 0.298 |
| Low (0-1) | 47 | 1.00 |  | 1.00 |  |
| High (2) | 22 | 2.48 (1.36-4.53) |  | 1.45 (0.72-2.93) |  |
| CD163 |  |  | <0.001 |  | 0.663 |
| Low (0-1) | 52 | 1.00 |  | 1.00 |  |
| High (2) | 19 | 3.05 (1.66-5.61) |  | 0.70 (0.14-3.57) |  |
| MARCO |  |  | 0.102 |  |  |
| Low (0-1) | 71 | 1.00 |  | ----- | ----- |
| High (2) | 2 | 3.33 (0.79-14.11) |  | ----- | ----- |
| Adjuvant treatment |  |  | 0.536 |  |  |
| Yes | 5 | 1.38 (0.50-3.85) |  | ------ | ----- |
| No | 70 | 1.00 |  | ------ | ----- |

Supplement 2: Hazard ratio for death according to clinicopathological factors, CD68^+^, CD163^+^ and MARCO^+^ macrophages in gastric cancer.
